# Correction: Prevalence and covariates of depression among older adults in Nepal: A systematic review and meta-analysis

**DOI:** 10.1371/journal.pmen.0000422

**Published:** 2025-08-29

**Authors:** Gayatri Khanal, Y. Selvamani, Suman Thapa, Saravanan Chinnaiyan

There is an error in affiliation 1 for authors Gayatri Khanal, Y. Selvamani and Saravanan Chinnaiyan. The correct affiliation 1 is: School of Public Health, SRM Institute of Science and Technology (SRMIST), Kattankulathur, Tamilnadu, India.
