# Supplementary material for: Prevalence and covariates of depression among older adults in Nepal: A systematic review and meta-analysis
Source: PLOS Ment Health. 2024 Sep 4;1(4):e0000112. doi: 10.1371/journal.pmen.0000112 (PMC12798329; doi:10.1371/journal.pmen.0000112)
Supplement: S2 File — (DOCX) [file pmen.0000112.s002.docx]

**S2 File: Search strategy**

P: Older /elder/geriatric population/senior citizens

Mesh (aged) or

Aged (mesh) or older (T/B) OR elder (t/b) OR Geriatric population or senior citizens

E: depression/ geriatric depression

Depression (Mesh) or depressive disorder (Mesh) or depression (All field ) or geriatric depression

**C**: Nepal

Pub med search

Concept 1: 3797164

**(((((aged [MeSH Terms])) OR (elder*[Title/Abstract])) OR (older [Title/Abstract])) OR ("senior citizen*"[Title/Abstract])) OR ("geriatric population*"[Title/Abstract])**

"aged"[MeSH Terms] OR "elder*"[Title/Abstract] OR "older"[Title/Abstract] OR "senior citizen*"[Title/Abstract] OR "geriatric population*"[Title/Abstract] =

Concept 2: 4,98,766

**(((depression [MeSH Terms]) OR (depressive disorder [MeSH Terms])) OR (depression [Title/Abstract])) OR (geriatric depression [Title/Abstract])**

"Depressive disorder"[MeSH Terms] OR "depression"[MeSH Terms] OR "depressive disorder"[MeSH Terms] OR "depression"[Title/Abstract] OR "geriatric depression"[Title/Abstract]

Concept 3: 15808

**(nepal[MeSH Terms]) OR (nepal[Title/Abstract])) OR (nepali[Title/Abstract])**

"nepal"[MeSH Terms] OR "nepal"[Title/Abstract] OR "nepali"[Title/Abstract]

**Merge of all concept: 67 articles**

**(((((((aged[MeSH Terms]) ) OR (elder*[Title/Abstract])) OR (older[Title/Abstract])) OR ("senior citizen*"[Title/Abstract])) OR ("geriatric population*"[Title/Abstract])) AND ((((depression[MeSH Terms]) OR (depressive disorder[MeSH Terms])) OR (depression[Title/Abstract])) OR (geriatric depression[Title/Abstract]))) AND (((nepal[MeSH Terms]) OR (nepal[Title/Abstract])) OR (nepali[Title/Abstract]))** Filters: **English, Humans, from 2013 - 2023**

(("aged"[MeSH Terms] OR "elder*"[Title/Abstract] OR "older"[Title/Abstract] OR "senior citizen*"[Title/Abstract] OR "geriatric population*"[Title/Abstract]) AND ("depressive disorder"[MeSH Terms] OR "depression"[MeSH Terms] OR "depressive disorder"[MeSH Terms] OR "depression"[Title/Abstract] OR "geriatric depression"[Title/Abstract]) AND ("nepal"[MeSH Terms] OR "nepal"[Title/Abstract] OR "nepali"[Title/Abstract])) AND ((humans[Filter]) AND (english[Filter]) AND (2013:2023[pdat]))
