# Supplementary material for: Prevalence and covariates of depression among older adults in Nepal: A systematic review and meta-analysis
Source: PLOS Ment Health. 2024 Sep 4;1(4):e0000112. doi: 10.1371/journal.pmen.0000112 (PMC12798329; doi:10.1371/journal.pmen.0000112)
Supplement: Attachment — Submitted filename: Response to Reviewer.docx [file pmen.0000112.s003.docx]

Dear Dr. Editor-in Chief

PLOS Mental Health

11/7/2024

Subject: Responding on peer reviewer and editor’s comments

We are pleased to submit our revised manuscript “**Prevalence and Covariates of Depression among Older Adults in Nepal: A Systematic Review and Meta-Analysis**” for your consideration that was given to the original version of the manuscript. After going through the suggestion of the reviewers and editor, we found out that most of the queries were rational and pertinent. From the reviewer, we got an opportunity to improve quality of our manuscript

We have addressed the issue raised by reviewer in the revision one by one and have addressed each issue below. Each of the co-authors has reviewed and approved of the revision. Please let us know if you have any additional queries.

We have attached two manuscripts. Manuscript 1 and manuscript 2

Manuscript 1 is labelled as “Revised Manuscript with highlight/Track Changes” where you can easily identify revision made because of highlight given which submitted as a separate file. Manuscript 2 is labeled as “Manuscript” which is unmarked or clean manuscript.

Lastly, our article would not have been better and near complete without the valuable comments from the reviewer and editors.

Thank you for the opportunity to revise this manuscript.

Warm Regards,

Gayatri Khanal

**Editor comments**

**Journal Requirements:**

1. If you did not receive any funding for this study, please simply state: “The authors received no specific funding for this work

**Response:** Issue addressed. Kindly see the highlighted declaration section at the end of manuscript.

**Reviewer One (R1)**

**Comment**

 I have reviewed the manuscript entitled "Prevalence and Covariates of Depression among Older Adults in Nepal: A Systematic Review and Meta-Analysis." The manuscript addresses a significant public health issue in a country with limited studies. While the study highlights the prevalence and covariates of depression among older adults in Nepal, several improvements can enhance its comprehensiveness and clarity. Despite the high heterogeneity among the studies evaluated, which undermines the reliability and generalizability of the findings, the study is valuable for understanding geriatric depression in Nepal.

**Response:** Thank you from bottom of our heart for appreciation and opportunity to improve quality of this manuscript. Now you can see the revised manuscript with a lots of scientific, technical and engligh improvements . If you feel manuscript still need revision, please let us know, we are happy to enhence the qulaity of our paper with your valauable suggestions.

**Comment 1**

**Introduction**
Various information was included early in the text, but the order may be more understandable. I recommend beginning the introduction with a concise and precise definition of depression. This should include its clinical characteristics, diagnostic criteria, and impact on daily life. Such a foundation will help readers understand the context of the study.
**Response:** Issue addressed. Paragraphs as well as entire manuscript modified as per the suggestions. Thank you.

Kindly refer to the introduction section of the manuscript.

**Comment 2**

**Introduction**

Highlight the specific ways in which depression manifests in older adults compared to younger individuals. Detail how symptoms may differ, why early detection is challenging, and the unique risk factors that older adults face. This differentiation is crucial for understanding why geriatric depression is a significant public health issue.

**Response:** Issue addressed. Highlighted the specific ways in which depression manifests in older adults, it’s symptoms, importance of early detection and unique risk factors among older people. Thank you.

Please refer to the highlighted introduction section of the manuscript.

**Comment 3**

**Introduction**

The authors may present statistical data on the prevalence of depression among older adults, particularly in developing countries. They should emphasize the lack of comprehensive data in such regions. This will underscore the importance of the current study and the need for updated epidemiological data.
Address the gaps in current research and the necessity for updated data on geriatric depression. Finally, explain the study's aim and how its findings can contribute to the literature.

**Response:** Issue addressed. Sentence has reframed highlighting the prevalence of depression in developing countries.

Please refer to highlighted introduction section in the manuscript.

**Comment 4**

**Discussion**

Discuss the geographic concentration of the studies included in the meta-analysis in detail. A significant proportion (60%) of these studies were conducted in the Central region of Nepal, particularly in the Kathmandu district. This concentration limits the generalizability of the findings to the entire country. It may not accurately reflect the prevalence of geriatric depression in other regions with different socio-economic and cultural contexts.

**Response:** Thank you for the comment. Limitation part of the discussion section has reframed with discussing this limitation in elaborative manner

Please refer to the limitation part of discussion section in the manuscript.

**Comment 5**

**Discussion**

It is necessary to discuss the types of questionnaires most commonly used to detect depressive mood disorders in the cross-sectional studies included in the analysis. The detail will provide insight into the methodologies employed and their potential impact on the results.

**Response:** Suggestion has incorporated in the discussion section. If you feel further revision. Please kindly let us know.

Please refer to the discussion section.

**Comment 6**

**Discussion**

Consider the potential confounding factors as a limitation of the study. While the paper identifies several risk factors for geriatric depression (e.g., female gender, chronic illness, lack of social support), it does not thoroughly address other factors that could influence these associations. For instance, socio-economic status, access to healthcare, and underlying health conditions might confound the relationships identified. A more detailed analysis considering these confounders would strengthen the study's conclusions.

**Response:** Since, this systematic review has done narrative synthesis in order to detect the risk factors hence we have added sentence like this “The present systematic review was included only observational studies’ evidence to detect the covariates of geriatric depression leads to contend with the possibility of confounding. Hence a more detailed analysis considering these confounders would strengthen the study’s findings.”

Apart from this entire manuscript has improvised as level best .

**Reviewer 2**

**Comment:** The article is important because of its relevance and filling the gap in our knowledge in global mental health area. It provided the evidence to implement further public health interventions and send strong message on developing the better services for people with depression.

**Response:** Thank you so much for your appreciation.

**Thank you!**
